# Plasma VEGF-D and sFLT-1 are potential biomarkers of hemodynamics and congestion in heart failure and following heart transplantation

**DOI:** 10.1016/j.jhlto.2023.100013

**Published:** 2023-10-30

**Authors:** Salaheldin Ahmed, Jakob Lundgren, Abdulla Ahmed, Göran Rådegran

**Affiliations:** a Department of Clinical Sciences Lund, The Section for Cardiology, Lund University, Lund, Sweden; b The Haemodynamic Lab, The Section for Heart Failure and Valvular Disease, VO. Heart and Lung Medicine, Skåne University Hospital, Lund, Sweden; c Department of Education and Research, Helsingborg Hospital, Helsingborg, Sweden

**Keywords:** heart failure, heart transplantation, pulmonary hypertension, right heart catheterization, tyrosine kinases

## Abstract

**Background:**

Inflammation and tyrosine-kinases are known mediators in the pathobiology of cardiovascular disease. Plasma biomarkers reflecting these systems may provide a noninvasive complement reflecting hemodynamics, aiding in clinical decision-making. We therefore aimed to investigate the plasma levels of vascular and inflammatory proteins, and their associations with invasive hemodynamics in advanced heart failure (HF) before, and at multiple follow-ups after heart transplantation (HT).

**Methods:**

Using multiplex sandwich immunoassays, absolute plasma concentrations of 9 vascular and inflammatory proteins were assessed in 26 patients with advanced HF, before HT, and at 4 weeks, 6 months, and 1year after HT. Right heart catheterization hemodynamics were assessed at the time of blood sampling. Repeated measures correlations were performed to evaluate the overall intra-individual development of plasma protein levels in relation to hemodynamics’ development over time.

**Results:**

Out of 9 proteins included initially, in advanced HF, elevated plasma levels of vascular endothelial growth factor D (VEGF-D) and soluble fms-like tyrosine kinase-1 (sFlt-1) decreased most markedly at 4 weeks (*p* < 0.0001), and decreased further at 6 months (*p* < 0.05) and at the 1 year follow-ups after-HT (*p* < 0.05). Over time, plasma VEGF-D correlated strongest with hemodynamic parameters including pulmonary arterial wedge pressure (PAWP) (*r*_mr_ = 0.75, 95% bootstrapped confidence interval (CI) 0.61-0.84, *p* < 0.0001), followed by mean right atrial pressure (MRAP) (*r*_mr_ = 0.74, 95% CI 0.61-0.82, *p* < 0.0001), and mean pulmonary arterial pressure (mPAP) (*r*_mr_ = 0.74, 95% CI 0.58-0.82, *p* < 0.0001). Plasma sFlt-1 correlated also with multiple hemodynamic parameters including PAWP (*r*_mr_ = 0.66, 95% CI 0.58-0.79, *p* < 0.0001), MRAP (*r*_mr_ = 0.64, 95% CI 0.58-0.81, *p* < 0.0001), and mPAP (*r*_mr_ = 0.61, 95% CI 0.51-0.76, *p* < 0.0001).

**Conclusions:**

In advanced HF, elevated plasma VEGF-D and sFlt-1 levels decrease early, already within 4 weeks after HT, and further throughout the first year postoperatively. Our findings support that high plasma VEGF-D and sFlt-1 concentrations before HT are related to congestion and overall hemodynamic improvement. Plasma VEGF-D and sFlt-1 may consequently be potential noninvasive biomarkers for monitoring hemodynamic deterioration and congestion in HF, and surveillance after HT.

Heart failure (HF) imposes a major clinical- and public health burden, globally affecting at least 26 million people.^1^ The heterogenous syndrome of HF is driven by a complex interplay of structural, functional, and hemodynamic perturbations, including capillary-stress failure, pulmonary congestion, and pericardial constraint.^2^, ^3^ The quality of life in HF remains poor, with unabated hospitalization rates and stagnating high mortality associated with the increased filling pressures and/or inadequate organ perfusion.^1^, ^3^

A frequent complication across the spectrum of HF phenotypes is pulmonary hypertension (PH), which arises due to sustained increase and backward transmission of left-sided filling pressures, further complicated by the superimposed components of pulmonary arterial vasoconstriction and endothelial dysfunction. Over the course of time, these perturbations may be followed by vascular remodeling of pulmonary venules and arterioles, and right ventricular dysfunction, with uncoupling of the “ventricular–arterial unit”, further imposing a worsening of the disease trajectory and prognosis.^4^

Although significant efforts and advances have been made in the management, treatment, and prevention of HF, the mechanistic understanding of this syndrome remains inadequate.^4^, ^5^ This is partly related to disparities in HF presentation, occurrence, and outcome, integrated with the aging population, frailty, genetics, and multimorbid burden, adding to the heterogeneity and complexity of this syndrome.^1^, ^3^, ^5^

Several pathways involved in maintaining and driving the progression of HF have been identified, including activation of the neurohormonal axis, inflammation, oxidative stress, myocardial injury, fibrosis, and matrix remodeling.^2^, ^6^ However, beyond the use of natriuretic peptides (NT-proBNP or BNP)—indicators of myocyte stress and right ventricular function, the clinical management in the European Society of Cardiology (ESC)/European Respiratory Society (ERS) guidelines of HF and PH have not yet endorsed the use of other biomarkers representative of other mechanisms.^4^, ^7^

Novel biomarkers, including proteomics, may represent and increase the understanding of the intricate interplay of the several mechanisms underlying HF and PH.^2^, ^6^, ^8^ These may complement traditional clinical-, laboratory-, and imaging modalities, potentially aiding in the diagnosis, prognosis, and monitoring of treatment response, thus allowing a more precise phenotyping, development of new therapies and individualized care, urged for in current ESC/ERS HF and PH guidelines.^4^, ^6^, ^7^

In previous studies, we investigated plasma inflammatory and vascular growth factors related proteins, with focus on tyrosine kinases, in the context of HF and related PH, before and 1 year after HT, using a relative quantification method.^9^, ^10^ We found several candidate biomarkers of prognostic and hemodynamic importance, including Interleukin (IL)-6 and vascular endothelial growth factor-D (VEGF-D), the latter appearing to be involved in lymphangiogenesis and possibly pulmonary congestion and vascular remodeling.^9^, ^10^, ^11^, ^12^

Biomarkers reflecting different processes recognized in HF, including hemodynamic derangements, may be of particular value in the management and follow-up of patients with advanced HF, allowing for earlier identification of clinical and hemodynamic deterioration, especially during the time of listing for, and after HT.^9^ Therefore, in the present study, we aimed to investigate the absolute concentrations of plasma inflammatory and vascular proteins, including tyrosine kinases, and their associations with invasive hemodynamics before, and at multiple follow-ups after HT.

## Material and methods

### Study population and blood sampling

The present study comprised 26 adult (≥18 years) patients with advanced HF, hemodynamically evaluated before HT, and at 4 weeks, 6 months, and 1 year after HT at Skåne University Hospital in Lund, Sweden. Patients with missing hemodynamics before HT at time of blood sampling (*n* = 3; due to implanted left ventricular assist device) or with PH after HT (*n* = 1) were excluded.

The study complies with the declarations of Helsinki and Istanbul. The study was approved by the local ethical board in Lund, Sweden (Dnr: 2010/114, 2010/442, 2011/368, 2011/777, 2014/92, and 2015/270).

Between November 2011 and May 2015, venous, nonfasting blood samples were collected prospectively from the introducers during the routine right heart catheterization (RHC) assessments prior to-, and at 4 weeks, 6 months, and 1 year after HT using vacutainer ethylenediaminetetraacetic acid tubes. Thereafter, the blood samples were centrifuged at 2000g at 20°C for 10 minutes and stored at −80°C in Lund Cardio Pulmonary Registry (LCPR), a prospective cohort in Region Skåne’s Biobank, initiated by Göran Rådegran in 2011.

### Protein analysis

Plasma samples were retrieved and analyzed using multiplex sandwich immunoassays (Meso Scale Discovery, Rockville, MD) to quantify the absolute plasma concentrations of 9 inflammatory and vascular proteins related to tyrosine kinase signaling, by using the commercially available kits at time of analysis. Each protein was measured in duplicates and the mean was calculated. Values with a coefficient of variation of ≥20 were excluded. The analyzed proteins were vascular endothelial growth factor A (VEGF-A), VEGF-D, soluble fms-like tyrosine kinase-1 (sFlt-1) or soluble VEGF receptor 1 (sVEGFR-1), angiopoietin-1 receptor (Tie2), placental growth factor (PlGF), fibroblast growth factor-2 (FGF-2), IL-6, IL-8, and tumor necrosis factor α (TNF-α).

### Clinical evaluation

Diagnosis and management of advanced HF, as well as the evaluation, and follow-up of HT were preformed according to prevailing guidelines by experienced cardiologists, at a national PH and HT referral center, at the Hemodynamic lab, at Skåne University Hospital in Lund, Sweden.^13^, ^14^ Cardiac transplantation procedures were performed at Skåne University Hospital in Lund, Sweden. Routinely, throughout the first year after HT, to monitor for acute cardiac allograft cellular rejection, surveillance endomyocardial biopsies were collected from the right interventricular septum and were subsequently histopathologically graded.^15^ According to prevailing guidelines at the time of clinical evaluation, PH due to left heart disease was defined hemodynamically by a mean pulmonary arterial pressure (mPAP) ≥25 mm Hg and pulmonary arterial wedge pressure (PAWP) >15 mm Hg. Moreover, postcapillary PH was further classified into either isolated postcapillary PH (pulmonary vascular resistance (PVR) ≤3 Wood units (WU)) or combined postcapillary and precapillary PH (PVR >3 WU).^16^

### Right heart catheterization and hemodynamic definitions

RHCs were performed at rest in supine position using a Swan Ganz catheter (Baxter Health Care Corp, Santa Ana, CA), inserted via an introducer into the right internal jugular vein, before HT, and at 4 weeks, 6 months, and 1 year after HT, at the hemodynamic lab, Skåne University Hospital in Lund, Sweden. Patients with elevated PVR underwent vasoreactivity testing with intravenous nitroprusside infusion, as a part of the hemodynamic evaluation before HT.

During RHC, thermodilution was performed to measure cardiac output (CO). Electrocardiography was used to determine heart rate (HR). Systolic and diastolic arterial pressures were measured noninvasively and the mean arterial pressure was calculated as 1/3 systolic +2/3 diastolic blood pressure. Directly obtained measurements included systolic- and diastolic mean pulmonary arterial pressures (sPAP, dPAP), mPAP, PAWP, mean right atrial pressure (MRAP). Additional parameters were calculated as following: cardiac index (CI) = CO/body surface area, stroke volume (SV) = CO × 1000/HR, SV index (SVI) = SV/body surface area, left ventricular stroke work index (LVSWI) = (mean arterial pressure − PAWP) × SVI, right ventricular stroke work index (RVSWI) = (mPAP − MRAP) × SVI, PVR = (mPAP − PAWP)/CO, total PVR= (mPAP/CO), pulmonary arterial compliance (PAC) = SV/(sPAP − dPAP).

In addition, during the RHC-procedure, the mixed venous oxygen saturation, arterial oxygen blood saturation, and arteriovenous oxygen difference were measured. Iohexol clearance was performed within a month before HT blood sampling and RHC. The creatinine-based estimate of glomerular filtration rate was calculated using the revised Lund-Malmö GFR estimating equation.^17^ Other clinical data were retrieved from the prospective LCPR-cohort file.

### Statistical analyses

The assumptions of normality were assessed visually with histograms. Nonparametric statistical tests were employed due to the dominance of non-Gaussian distributed data. Continuous data were presented as median (inter quartile range (IQR)). Two-sided Wilcoxon’s signed-rank tests were performed to assess the mean rank-difference between paired groups. To adjust the threshold of statistical significance, correction with false discovery rate (FDR) was used, calculated using the 2-stage step-up method of Benjamini, Krieger, and Yekutieli. Kaplan Meier analysis was employed to describe the survival and all-cause mortality after HT. To evaluate the trajectory of plasma protein levels in relation to RHC hemodynamics’ development over time after HT, repeated measures correlations were conducted. The effect sizes were estimated using bootstrapped confidence intervals of the correlation coefficients, which were calculated due to non-normally distributed data.

Statistical analyses were conducted using R version 4.2.2 (R Core Team, 2022), RStudio (Rstudio Team, 2022), including the rmcorr package,^18^ as well as GraphPad Prism version 9.5.0 for Windows (GraphPad Software, San Diego, CA).

### Study setup

Proteins with significant plasma level dynamics in comparison to the levels before HT, that is (high or low levels at baseline, followed by an increase, decrease, or plateau after HT), after correction with FDR across different groups, were eligible for correlation analysis. Further nonparametric testing between the other groups (4 weeks vs 6 months and 1 year, and 6 months vs 1 year) was conducted as indicated to further investigate plasma dynamics. Proteins with the strongest correlation coefficients were of particular focus, and in relation to previous publications.^9^, ^10^

## Results

### Population characteristics and survival

**Figure 1:**
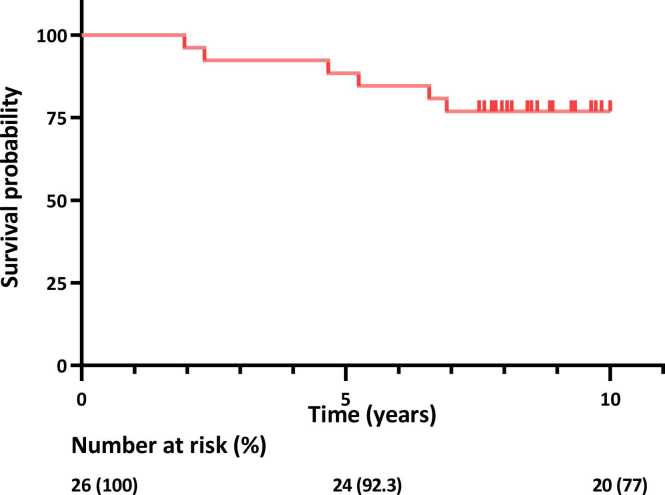
Survival of patients with advanced heart failure at inclusion, following heart transplantation, censoring was done at 10 years of follow-up.

The demographics of the study population at baseline are described in Table 1. The median listing time for HT was 98.5 (64-168) days. During the study follow-up period between November 11, 2011, and May 30, 2022, corresponding to a median follow-up time of 8.1 (7.5-9.2) years, 6 patients (23.1%) died. Of those, 2 patients (7.7%) died within 3 years, another 2 (7.7%) died within 5 years, and additional 2 (7.7%) died within 7 years after HT (Figure 1). Patients’ hemodynamics at baseline and follow-up assessments after HT are listed in Table 2. Primary graft failure within the first 24 hours after HT requiring short-term extracorporeal membrane oxygenation support occurred in 1 patient (3.4%). Infections requiring hospital admission, prolonged hospital stay, or intravenous treatments occurred at least once in 12 patients (46.2%). Surveillance endomyocardial biopsies revealed that mild, asymptomatic rejections (1R≤) occurred in 8 patients (30%) at 4 weeks, in 6 (24%) at 6 months, and in 8 (32%) at 1 year follow-ups after HT. Acute cellular ejections graded ≥2R were not observed at 4 weeks, 6 months, nor at the 1 year follow-up after HT. Clinical symptoms indicative of rejection were not experienced by any of the patients during blood sampling and the hemodynamic assessments.

**Table 1:** Patients’ Demographics at Baseline

Parameter	*n* (26)	Values
Female, *n* (%)	26	7 (26.9)
Age (years)	26	50.0 (42.25-60.0)
Age at transplantation (years)	26	50.0 (44.25-60.25)
Weight (kg)	26	81.0 (70.1-93.0)
Length (cm)	26	177.0 (172.0-181.0)
LVEF (%)	24	25.0 (10.0-27.5)
Peak-VO_2_ (ml/kg/min)	20	11.9 (10.2-13.8)
Pre-HT LVAD, *n* (%)	26	11 (42)
PH classification		
PH-LHD, *n* (%)	24	19 (79.2)
Ipc-PH, *n* (%)	19	11 (57.9)^a^
Cpc-PH, *n* (%)	19	7 (36.8)
Heart failure etiology		
DCM, *n* (%)	26	19 (73.1)
HCM, *n* (%)	26	2 (7.7)
RCM, *n* (%)	26	1 (3.8)
IHD, *n* (%)	26	3 (11.5)
Re-Tx, *n* (%)	26	1 (3.8)
Comorbidities		
History of smoking, *n* (%)	25	7 (28)
Diabetes mellitus, *n* (%)	25	2 (8)
Hypertension, *n* (%)	26	3 (11.5)
Stroke, *n* (%)	26	2 (7.7)
Atrial fibrillation *n* (%)	26	7 (26.9)
Medications		
Beta blockers, *n* (%)	26	26 (100)
ACEi/ARB, (%)	26	11/11 (81)
MRA, (%)	26	20 (77)
Diuretics, (%)	26	26 (100)
Recent levosimendan	24	5 (21)
Biochemical indicators		
NT-proBNP, (ng/liter)	21	4126 (3663-7306)
P-creatinine, (µMol/liter)	26	106 (91-135)
eGFR-creatinine (ml/min/1.73 m^2^)	26	62.4 (53.7-71.0)
Iohexol GFR, (ml/min/1.73 m^2^)	23	61 (46-74.5)

Abbreviations: ACEi, angiotensin converting enzyme inhibitor; ARB, angiotensin II receptor blocker; Cpc-PH, combined post and precapillary PH; DCM, dilated cardiomyopathy; eGFR, estimated glomerular filtration rate; HCM, hypertrophic cardiomyopathy; IHD, ischemic heart disease; Ipc-PH, isolated post-capillary PH; LVAD, left ventricular assist device; MRA, mineralocorticoid receptor antagonist; PH, pulmonary hypertension; PH-LHD, PH due to left heart disease.

The values are described as median (interquartile range), unless otherwise stated.

a One patient suffered from severe orthopnea during right heart catheterization (RHC) and pulmonary arterial wedge pressure could therefore not be assessed. Subsequent hemodynamic assessment revealed Ipc-PH.

**Table 2:** Patients’ Hemodynamics Before and at Multiple Follow-ups After Heart Transplantation

Parameter	Before HT		4 weeks after HT		6 months after HT		1 year after HT	
	*n* (26)	Median (IQR)	*n* (24)	Median (IQR)	*n* (26)	Median (IQR)	*n* (17)	Median (IQR)
BSA (m^2^)	26	2 (1.8-2.2)	22	1.9 (1.8-2.1)	24	1.9 (1.8-2.1)	17	2 (1.9-2.1)
MAP (mm hg)	24	83 (78-94)	22	93 (85-100)	24	103 (98-115)	17	105 (97-115)
mPAP (mm hg)	24	31 (25-39)	22	18 (12-21)	24	17 (12-19)	17	16 (13-21)
TPG (mm hg)	24	9 (6-11)	22	7 (6-9)	24	9 (6-10)	17	8 (6.5-11)
PAWP (mm hg)	23	20 (18-27)	22	9.5 (5-11)	24	7 (5-10)	17	6 (5-9.5)
MRAP (m mhg)	24	15 (9-18)	22	4.5 (3-6)	24	2.5 (1-4)	17	2 (0.5-4.5)
HR (beats/min)	24	73 (69-85)	22	85 (76-90)	24	78 (73-83)	17	77 (65-88)
CO (liter/min)	24	3.2 (2.6-4)	22	5.9 (5.4-6.7)	24	5.5 (5.1-6)	16	5.3 (5-6.1)
CI (liter/min/m^2^)	24	1.6 (1.4-.9)	22	2.9 (2.8-3.4)	24	3 (2.6-3.2)	16	2.7 (2.5-3.2)
SV (ml/beat)	24	45 (34-57)	22	71 (64-80)	24	74 (65-80)	16	75 (68-81)
SVI (ml/beat/m^2^)	24	24 (18-28)	22	36 (32-40)	24	38 (34-42)	16	37 (35-40)
LVSWI (mm Hg × ml/m^2^)	23	1255 (1095-1907)	22	2906 (2700-3419)	24	3690 (3280-4183)	16	3567 (3224-4157)
RVSWI (mm Hg × ml/m^2^)	24	373 (268-596)	22	440 (292-535)	24	497 (416-645)	16	471 (340-739)
a-vO_2_diff (ml O_2_/liter)	24	74 (66-88)	15	43 (40-49)	20	46 (42-49)	14	45 (39-49)
SaO_2_ (%)	24	95 (94-96)	15	97 (94-98)	20	96 (95-98)	14	96 (94-97)
SvO_2_ (%)	24	51 (45-60)	22	66 (62-68)	24	68 (64-71)	17	69 (65-71)
PVR (WU)	23	2.5 (2.1-3.5)	22	1.3 (0.96-1.5)	24	1.5 (1.1-1.9)	16	1.7 (1.2-2.1)
PVRI (WU/m^2^)	23	5.3 (3.5-6.8)	22	2.3 (1.9-3.1)	24	2.9 (2-3.5)	16	3.2 (2.4-4.2)
TPVR (WU/m^2^)	24	11 (6.8-12)	22	2.7 (1.8-3.5)	24	2.7 (2.2-3.5)	16	2.8 (2-4.1)
SVR (WU)	24	21 (19-26)	22	14 (12-17)	24	18 (16-20)	16	20 (17-21)
PAC (ml/mm Hg)	24	2.3 (1.8-3.5)	21	5.3 (4-6.9)	24	4.7 (3.5-6.4)	16	4.9 (3.8-6.2)

Abbreviations: a-vO_2_diff, arteriovenous oxygen difference; BSA, body surface area; CI, cardiac index; CO, cardiac output; HR, heart rate; IQR, interquartile range; LVSWI, left ventricular stroke work index; MAP, mean arterial pressure; mPAP, mean pulmonary arterial pressure; MRAP, mean right atrial pressure; PAC, pulmonary arterial compliance; PAWP, pulmonary arterial wedge pressure; PVR, pulmonary vascular resistance; PVRI, pulmonary vascular resistance index; RVSWI, right ventricular stroke work index; SaO_2_, arterial oxygen saturation; SV, stroke volume; SVI, stroke volume index, SvO_2_, venous oxygen saturation; SVR, systemic vascular resistance; TPG, transpulmonary pressure gradient; TPVR, total pulmonary vascular resistance; WU, wood units.

### High plasma VEGF-D and sFlt-1 decrease most markedly in the first 4 weeks after heart transplantation

**Figure 2:**
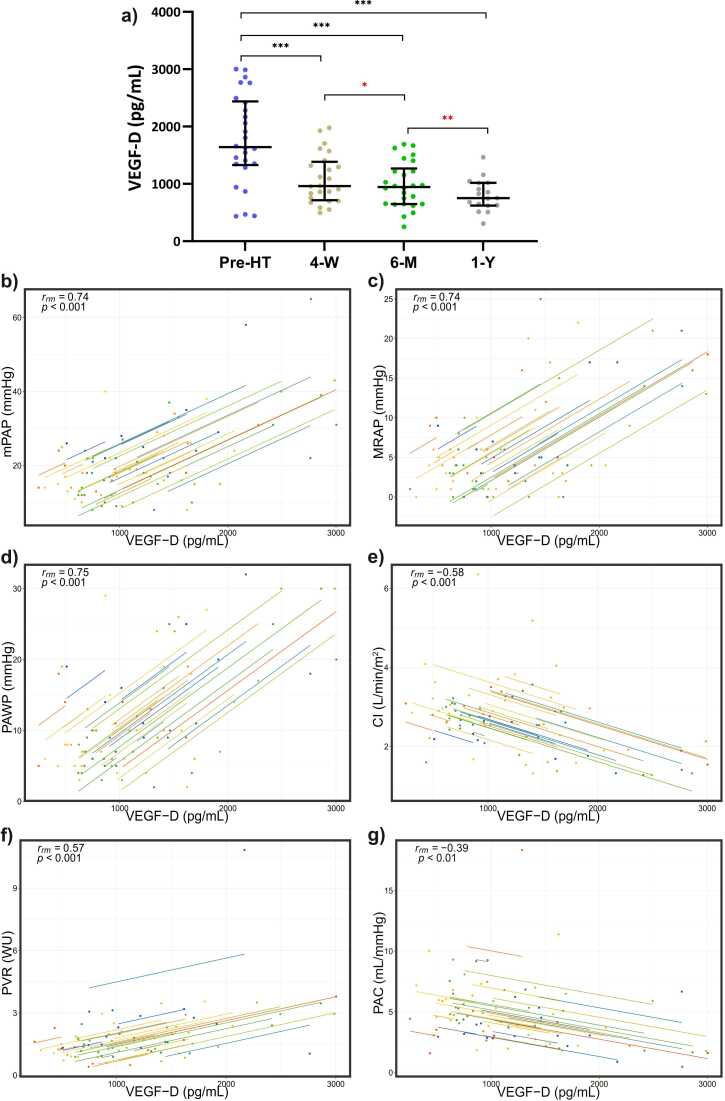
Plasma VEGF-D levels **(a)**, and **(b-g)** repeated measures correlation analyses with multiple hemodynamic parameters before and at multiple follow-ups after heart transplantation (HT). Comparisons between groups **(a)** were done with Wilcxon signed rank tests. **(b-g)** Each point represents a certain follow-up with simultaneously collected blood sample. Each line represents a single patient. The correlation coefficient (r_rm_) indicate the resultant (overall) direction of intra-individual correlation. Abbreviations: 4-W; 4 weeks follow-up after HT; 6-M, 6 months follow-up after HT; 1-Y, 1 year follow-up after HT; CI, cardiac index; mPAP, mean pulmonary arterial pressure; MRAP, mean right atrial pressure; PAC, pulmonary arterial compliance; PAWP, pulmonary arterial wedge pressure; PVR, pulmonary vascular resistance; Pre-HT, prior to HT; VEGF-D, vascular endothelial growth factor D; WU, wood units. ***p-value < 0.001; **p-value < 0.01, *p-value < 0.05. False discovery rate (Q = 1%) was applied on all comparisons, excluding comparisons indicated with red asterisk (posthoc analysis) in which a *p*-value of 0.05 was considered statistically significant.

**Figure 3:**
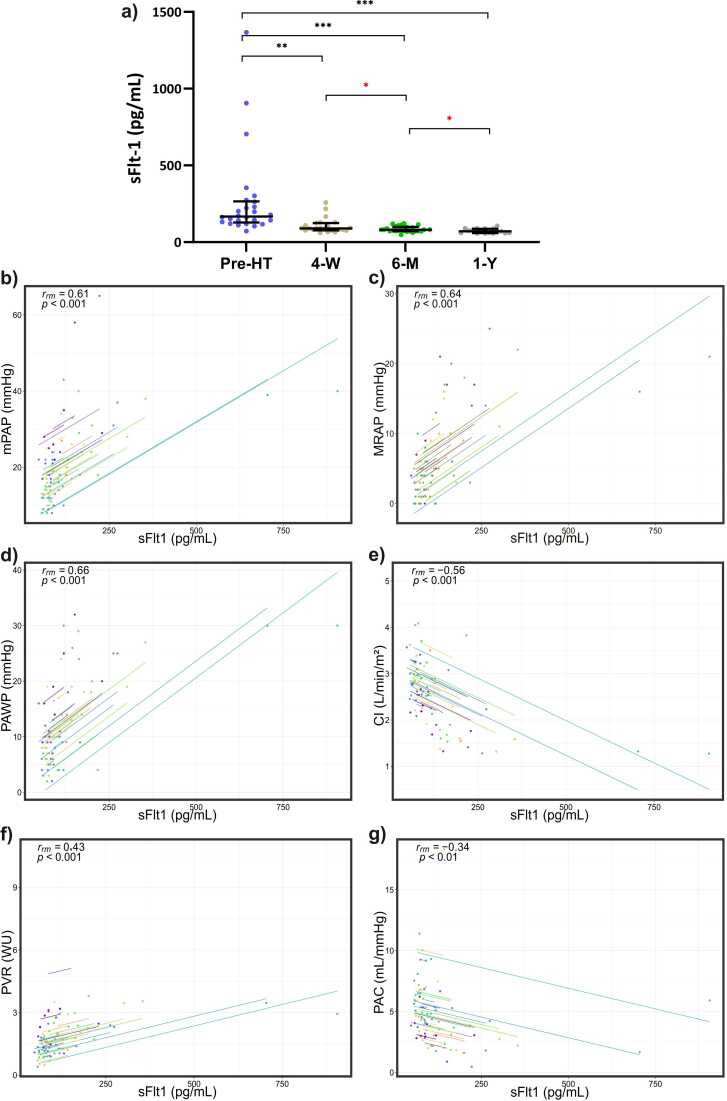
Plasma sFlt-1 levels **(a)**, and **(b-g)** repeated measures correlation analyses with multiple hemodynamic parameters before and at multiple follow-ups after heart transplantation (HT). Comparisons between groups **(a)** were done with Wilcxon signed rank tests. **(b-g)** Each point represents a certain follow-up with simultaneously collected blood sample. Each line represents a single patient. The correlation coefficient (*r*_rm_) indicates the resultant (overall) direction of intra-individual correlation. Abbreviations: 4-W, 4 weeks follow-up after HT; 6-M, 6 months follow-up after HT; 1-Y, 1 year follow-up after HT; CI, cardiac index; mPAP, mean pulmonary arterial pressure; MRAP, mean right atrial pressure; PAC, pulmonary arterial compliance; PAWP, pulmonary arterial wedge pressure; Pre-HT, prior to HT; PVR, pulmonary vascular resistance; sFlt-1, soluble fms-like tyrosine kinase-1; WU, wood units. ***p-value < 0.001; **p-value < 0.01, *p-value < 0.05. False discovery rate (Q = 1%) was applied on all comparisons, excluding comparisons indicated with red asterisk (posthoc analysis) in which a *p*-value of 0.05 was considered statistically significant.

Plasma levels of proteins preoperatively and at the follow-ups after HT are presented in Table 3. In advanced HF, high plasma VEGF-D and sFlt-1 levels decreased most markedly at the 4 weeks follow-up, as well as vs 6 months and 1 year after HT (FDR = 1%, *p* < 0.01, all groups vs before-HT; Figures 2 and 3). VEGF-D and sFlt-1 continued to decrease throughout the first year postoperatively (4 weeks vs 6 months, 4 weeks vs 1 year and 6 months vs 1 year after HT (*p* < 0.05, Figures 2 and 3).

**Table 3:** Absolute Plasma Levels of Vascular and Inflammatory Proteins Before and at Multiple Follow-ups After Heart Transplantation

Protein, pg/ml	Before HT (*n* = 26)	4 weeks after HT (*n* = 24)	6 months after HT (*n* = 26)	1 year after HT (*n* = 17)	*p*-values		
	Median (IQR)	Median (IQR)	Median (IQR)	Median (IQR)	Before vs 4 week post HT	Before vs 6 months post HT	Before vs 1 year post HT
FGF-2	21 (10-36)	19 (9.4-36)	13 (8.6-32)	11 (7.2-19)	0.97	0.52	0.21
IL-6	1.3 (1-2.6)	2.4 (1.2-3.5)	0.75 (0.53-1.5)	0.74 (0.47-0.97)	0.037	0.046	0.00021^a^
IL-8	5.8 (4-8.3)	3.9 (2.5-5.5)	5.2 (3.6-7.4)	3.7 (3.3-5.2)	0.016	0.99	0.02
*PlGF*	*26 (18-33)*	*39 (33-44)*	*36 (29-42)*	*31 (30-35)*	*0.000091*^a^	*0.000095*^a^	*0.031*
*sFlt-1*	*167 (129-265)*	*89 (77-124)*^b^	*80 (72-99)*	*70 (59-87)*	*0.0003*^a^	*8.9* *×* *10^−8^*^a^	*0.000015*^a^
*Tie2*	*3825 (3243-4224)*	*3278 (2877-3638)*	*2934 (2796-3413)*	*2999 (2766-3488)*^b^	*0.0012*^a^	*0.00028*^a^	*0.018*
TNF-α	2.6 (2.3-3.9)	3 (2-5.8)	2.7 (2.4-3.3)	3 (2.3-3.4)	0.56	0.58	0.82
VEGF-A	72 (53-131)^b^	115 (77-132)	111 (73-129)	75 (66-101)	0.17	0.17	0.35
*VEGF-D*	*1643 (1330- 2437)*	*960 (715-1385)*	*945 (648-1268)*	*750 (623-1018)*	*0.000025*^a^	*2.6* *×* *10^−6^*^a^	*0.000031*^a^

Abbreviations: FGF-2, fibroblast growth factor-2; IL, interleukin; IQR, interquartile range; PlGF, placental growth factor; sFlt-1, soluble fms-like tyrosine kinase-1 or soluble VEGF receptor 1 (sVEGFR-1); Tie2, angiopoietin-1 receptor; TNF-α, tumor necrosis factor α; VEGF-A, vascular endothelial growth factor A.

a Indicates a statistically significant difference (*p* < 0.007, false discovery rate = 1%). The rows of significant proteins in all 3 comparisons are highlighted in italics.

b Indicates (*n* − 1).

### Repeated measures correlations—VEGF-D correlates strongest with PAWP

Repeated measures correlation analyses of VEGF-D and sFlt-1 with hemodynamic parameters are presented in Table 4. Plasma VEGF-D and sFlt-1 correlated with multiple hemodynamic variables over time following HT. VEGF-D correlated intra-individually strongest with PAWP (*r*_mr_ = 0.75, bootstrapped 95% confidence interval (CI) 0.61-0.84, *p* < 0.0001), followed by mPAP (*r*_mr_ = 0.74, 95% CI 0.58-0.82, *p* < 0.0001), and MRAP (*r*_mr_ = 0.74, 95% CI 0.61-0.82, *p* < 0.0001), Figure 2, Table 4. Plasma sFlt-1 correlated strongest with PAWP (*r*_mr_ = 0.66, 95% CI 0.58-0.79), followed by MRAP (*r*_mr_ = 0.64, 95% CI 0.58-0.81, *p* < 0.0001), and mPAP (*r*_mr_ = 0.61, 95% CI 0.51-0.76, *p* < 0.0001), Figure 3, Table 4.

**Table 4:** Repeated Measures Correlations of Significant Plasma Proteins and Hemodynamics Before- and After Heart Transplantation

Protein (pg/ml)	mPAP (mm hg)	PAWP (mm Hg)	MRAP (mm Hg)	CI (liter/min/m^2^)	PVR (WU)	PAC (ml/mm Hg)
	*r*_mr_ (95% CI, *p*-value)	*r*_mr_ (95% CI, *p*-value)	*r*_mr_ (95% CI, *p*-value)	*r*_mr_ (95% CI, *p*-value)	*r*_mr_ (95% CI, *p*-value)	*r*_mr_ (95% CI, *p*-value)
sFlt-1	0.61 (0.51-0.76, 2.4 × 10^−7^)^a^	0.66 (0.58-0.79, 1.0 × 10^−8^)^a^	0.64 (0.58-0.81, 3.9 × 10^−8^)^a^	−0.56 (−0.76 to −0.48, 3.5 × 10^−6^)^a^	0.43 (0.33-0.72, 0.00062)^a^	−0.34 (−0.51 to −0.19, 0.008)^a^
PlGF	−0.44 (−0.59 to −0.29, 0.00032)^a^	−0.43 ( −0.62 to −0.28, 0.00064)^a^	−0.5 (−0.66 to −0.31, 0.000032)^a^	0.46 (0.24-0.65, 0.0002)^a^	−0.43 (−0.57 to −0.35,0.00056)^a^	0.18 (−0.02 to 0.4, 0.18)
Tie2	0.4 (0.14-0.6, 0.001)^a^	0.29 (0.08-0.48, 0.024)^a^	0.35 (0.15-0.51, 0.006)^a^	−0.41 (−0.56 to −0.17, 0.001)^a^	0.32 (0.065-0.48, 0.014)^a^	−0.014 (−0.33 to −0.26, 0.92)
VEGF-D	0.74 (0.58-0.82, 7.1 × 10^−12^)^a^	0.75 (0.61-0.84, 5.3 × 10^−12^)^a^	0.74 (0.61-0.82, 8.0 × 10^−12^)^a^	−0.58 (−0.74 to −0.42, 9.3 × 10^−7^)^a^	0.57 (0.43-0.76, 1.9 × 10^−6^)^a^	−0.39 (−0.59 to −0.24, 0.002)^a^

Abbreviations: CI, bootstrapped confidence interval; CI, cardiac index; mPAP, mean pulmonary arterial pressure; MRAP, mean right atrial pressure; PAC, pulmonary arterial compliance; PAWP, pulmonary arterial wedge pressure; PlGF, placental growth factor; PVR, pulmonary vascular resistance; *r*_mr_, repeated measures correlation coefficient; sFlt-1, soluble fms-like tyrosine kinase-1 or soluble VEGF receptor 1 (sVEGFR-1); Tie2, angiopoietin-1 receptor; VEGF-D, vascular endothelial growth factor D; WU, wood units.

a Indicates a statistically significant difference (*p* < 0.05, false discovery rate = 1%).

### Plasma Tie2 and PlGF correlated with hemodynamics

In advanced HF, high plasma Tie2 levels decreased after HT at 4 weeks and 6 months vs before HT, (*p* < 0.01, FDR = 1%), whereas low plasma PlGF preoperatively increased at 4 weeks and 6 months vs before HT (*p* < 0.0001, FDR = 1%), Table 3. Both Tie2 and PlGF showed a tendency toward lower and higher levels, respectively, at the 1 year-follow-up after vs before HT, Table 3. Both Tie2 and PlGF levels correlated moderately with multiple hemodynamic variables over time, Table 4.

## Discussion

Invasive evaluation using RHC remains the gold standard for monitoring hemodynamics in advanced HF, as well as in the assessments for left ventricular assist device and maintaining cardiac transplant candidacy.^19^, ^20^ The latest guidelines of the International Society for Heart and Lung Transplantation recommend preoperative and periodic hemodynamic surveillance with individualized frequency; and highlight the incremental value of RHC, especially in cases of clinical deterioration, postgraft PH, and acute cardiac allograft rejection.^19^, ^21^

Several attempts have been made to characterize a multitude of biomarkers.^2^, ^6^ However, thus far, beyond their use in the management of HF, PH, and surveillance in relation to HT, natriuretic peptides and troponins remain the only markers largely utilized in routine clinical practice.^4^, ^7^, ^20^, ^22^ In the present study, we investigated the absolute concentrations of plasma proteins related to inflammation and tyrosine kinase signaling in reflecting invasive hemodynamic changes before- and at multiple follow-ups after HT. Out of 9 proteins initially included, VEGF-D and sFlt-1 were the most promising, declining after HT and correlating the strongest with invasive hemodynamics, including parameters of pulmonary vascular congestion and ventricular function, emerging as potential biomarker candidates for surveillance after HT.

PH is an established complicating factor during listing for HT and after HT, with negative impact on prognosis.^20^, ^23^ In the present study, high plasma VEGF-D and sFlt-1 levels correlated intra-individually the strongest with PAWP, and MRAP, estimates of left- and right ventricular filling pressures, as well as with mPAP, CI, PAC, and PVR. However, the correlations of PAC and PVR may be a direct result of decongestion, possibly explained by the inverse hyperbolic PVR and PAC relationship which is sensitive to PAWP changes,^24^ as well as improved CO and SVI after HT. Nevertheless, plasma biomarkers may be of huge importance, offering a noninvasive complement to RHC in monitoring disease progression, along with clinical and imaging modalities. This should, however, be investigated in further studies, focusing on whether the plasma levels are a cause or consequence of HF, as well as on temporal/situational aspects (acute decompensation vs optimally treated patients) in assessing VEGF-D and sFlt-1 levels, particularly as treatment with diuretics impact right- and left-sided filling pressures.^25^, ^26^

VEGF-D is an essential factor in regulating lymphangiogenesis through binding to VEGFR-3, but also in regular angiogenesis, cardiac remodeling, and endothelial proliferation through binding to VEGFR-2/3.^27^, ^28^, ^29^ VEGF-D is a secreted glycoprotein, most abundantly expressed in the lungs, and together with VEGF-C and their receptor VEGFR-3, exert important biological functions in the maintenance of lymphatic vessels including lymphatic sprouting and drainage as well as homeostasis of tissue fluid.^30^, ^31^ In lymphangioleiomyomatosis, plasma VEGF-D is used as a diagnostic and a severity marker.^32^ Elevated levels of VEGF-D have been described in patients with atrial fibrillation and ischemic stroke,^33^ HF,^9^ congestion,^11^, ^12^ and PH.^34^, ^35^ In our previous study, elevated plasma VEGF-D in advanced HF decreased 1 year after HT toward healthy controls levels, and correlated inter-individually with multiple invasive hemodynamic parameters, including PAC,^12^ which is a prognostic marker for HF and PH,^36^ as well as a predictor of mortality in HF with preserved ejection fraction.^37^ In the present study, VEGF-D correlated over time with changes in PAC. However, due to the low number of patients, prognostic analysis could not be conducted. Recently, in a large-scale study including a discovery and a validation cohort, both circulating and genetic variants of VEGF-D were found to be independently associated with increased cardiovascular mortality in patients with acute and chronic coronary syndrome.^38^ Overall, as previously proposed, VEGF-D elevation may be a direct result of congestion, and act to alleviate congestion symptoms by increasing lymphangiogenesis and lymphatic clearance.^9^

While VEGF-D/VEGFR-3 binding may be crucial in lymphangiogenesis, VEGF-A is an essential factor of angiogenesis, and through binding to its receptors Flt-1 (also known as VEGFR-1) and/or VEGFR-2 regulate a multitude of different mechanisms including endothelial cell migration, proliferation, vascular permeability, and secretion.^39^ The soluble form of VEGFR-1, sFlt-1, is suggested to be important in the negative regulation of angiogenesis through acting as a decoy receptor, trapping VEGFs, and PlGF.^39^ PlGF is expressed in various cell types and tissues, including the placenta, heart and lungs, promoting endothelial cell survival and angiogenesis.^39^, ^40^ Elevated sFlt-1 levels have been reported in preeclampsia, correlating with the degree of the disease, inducing hypertension, by inhibiting VEGF-mediated endothelial NO production,^39^ as well as in different forms of PH, and in HF, with potential impact on vascular remodeling.^40^, ^41^ In patients with HF, although sFlt-1 levels correlated with the disease severity and were prognostic of death, cardiac transplantation, or implantation of ventricular assist device, enhancing the sole predictive value of BNP, plasma PlGF failed to be an independent predictor of disease severity or outcomes.^40^, ^42^ Moreover, elevated sFlt-1 levels are associated with myocardial injury^43^ and predicts incident HF and cardiovascular mortality,^44^ whereas high plasma levels PlGF are thought to promote endothelial healing and stimulate microvascular angiogenesis after myocardial infarction, improving outcomes.^43^, ^45^ In a previous study, we found higher plasma levels of PlGF in advanced HF and in the 1 year follow-up after HT vs healthy controls, but not before vs after HT.^9^ The present study confirms these findings, adding that PlGF levels are the highest early after HT, and decrease throughout the first 6 months after HT. Also, higher plasma levels correlated with improved hemodynamics over time, particularly with MRAP. Plasma PlGF may, however, not be a good indicator of hemodynamics due to the absence of a plasma level change before vs 1 year after HT (Tables 3 and 4). Moreover, our findings of sFlt-1 and its correlations with hemodynamics indicate that it may be related to the pathogenesis of HF with PH, including the vascular remodeling,^46^ in line with the abovementioned studies.

Important complications following HT include early graft dysfunction, cardiac allograft vasculopathy, and acute allograft rejection, the latest accounting for 5% of deaths within the first month after HT.^42^ Symptoms of low cardiac output and congestion may indicate acute cardiac allograft rejection.^20^ Impaired cardiac contractility, and consequently increased cardiac filling pressures along with activated neuro-hormonal axis and interstitial fluid accumulation, comprise the currently proposed main mechanism in which congestion is induced and perpetuated in HF.^22^, ^47^ Additional contributing mechanisms include inflammation, increased vascular permeability, and vascular interstitial leakage as well as endothelial dysfunction, such as in PH.^22^, ^47^, ^48^ Acute cardiac allograft rejection remains diagnostically challenging due to occurrences in asymptomatic patients, significant histopathological inter-observer variability and low yield for detecting moderate or higher severity rejection using surveillance endomyocardial biopsies.^20^ Despite these limitations, novel approaches aiding in further classification, surveillance, and detection of allograft rejection are emerging including assessment of rejection-associated gene transcripts and biomarkers.^20^ In the present study, high plasma VEGF-D and sFlt-1 levels decreased most markedly already within the 4 weeks follow-up assessments (*p* < 0.0001) and continued to decrease further at the 6 months and at the 1 year follow-up assessments after HT. Apart from correlating with multiple invasive hemodynamic parameters, the strongest being with PAWP, our results support the notion that VEGF-D and sFlt-1 may be markers of improved overall hemodynamics including cardiac function, but primarily congestion.^9^, ^11^, ^12^, ^38^

Tie2 exert different cellular effects depending on the biding substrate. For instance, binding of Tie2 to its agonistic ligand angiopoietin-1 results in maintaining the quiescence and integrity of endothelial cells, whereas binding to angiopoietin-2, a partial Tie2 antagonist or agonist, modulate angiopoinetin-1/Tie2 signaling in an autocrine-, organ-, and context-specific manner, promoting destabilization of endothelial integrity in hypoxia, and augmenting vascular permeability and inflammation.^49^, ^50^ We have previously demonstrated that elevated plasma Tie2 levels decreased and normalized toward healthy controls’ levels at the 1 year follow-up after HT, but did not correlate with the improved hemodynamic parameters after HT.^9^ In the present study, we confirm the presence of high plasma Tie2 levels in advanced HF and add that the decrease is most markedly during the first 6 months after HT. Although having the potential to be a marker of hemodynamic improvement after HT due to displaying moderate intra-individual correlations with hemodynamics over time, further studies are needed to elaborate its clinical potential in relation to its ligands’ angiopoietin 1 and 2.

Strengths of the present study include the invasive hemodynamic assessments, the prospectively collected blood samples, and the well-defined study population with a long-term follow-up. Repeated measures correlations analysis assesses the linear and overall intra-individual relationship of paired data without violating independence assumptions, but does not assess curvilinear relationships.^18^ A limitation is the relatively small study population, which however, corresponds with previous HT studies that resulted in larger trials, and can be partly explained by the single center nature.^9^ Importantly, although measured at different timepoints, the over time changes of the protein levels together with the correlations do not imply causality, and the hemodynamic improvement after HT may not necessarily be directly related to the biomarkers. Larger, multicenter studies are encouraged to investigate the role of plasma VEGF-D and sFlt-1 at different timepoints in relation to HF, PH, and HT. Although the participants may act as their own controls, adjustment for the impact of immunosuppressive medication and investigating the biomarkers’ potential incremental values to natriuretic peptides, and troponins need to be addressed in the future.

## Conclusions

To our knowledge, this is the first study to investigate plasma VEGF-D and sFlt-1 levels before and at multiple follow-ups throughout the first year after HT. Over time, plasma VEGF-D and sFlt-1 correlated intra-individually with multiple invasively measured hemodynamics, including MRAP and PAWP, supporting the notion that VEGF-D and sFlt-1 may be involved in vascular remodeling and/or congestion. Plasma VEGF-D and sFlt-1 may provide noninvasive complements reflecting invasive hemodynamics, aiding in clinical decision-making in HF, and in surveillance after HT, in which RHC may be considered.

## CRediT authorship contribution statement

Salaheldin Ahmed: conceptualization, methodology, software, validation, investigation, writing – original draft, formal analysis, visualization. Jakob Lundgren: conceptualization, methodology, visualization, investigation, writing – review and editing. Abdulla Ahmed: conceptualization, methodology, visualization, investigation, writing – review and editing. Göran Rådegran: conceptualization, methodology, investigation, resources, writing – review and editing, supervision, project administration, and funding acquisition. All authors are accountable for all aspects of the work and approve of the final version to be published.

## Funding

The work was supported by research grants from “Avtal om Läkarutbildning och Forskning” (ALF), “Skånes University hospital foundations and donations” and Go Rad Care AB. The funding organizations played no role in the collection, analysis, or interpretation of the data and had no right to restrict the publishing of the manuscript.

## Disclosure statement

The authors declare the following financial interests/personal relationships which may be considered as potential competing interests: Dr Salaheldin Ahmed reports financial support was provided by Go Rad Care AB. Dr Göran Rådegran reports financial support was provided by Avtal om läkarutbildning och forskning (ALF). Dr Salaheldin Ahmed reports a relationship with Janssen Cilag AB and Nortic Infucare that includes consulting or advisory. Dr Abdulla Ahmed reports a relationship with Janssen Cilag AB and Nortic Infucare that includes consulting or advisory. Dr Jakob Lundgren reports a relationship with Janssen Cilag AB, Orion Pharma AB, Astra Zeneca AB, and Bristol Myers Squibb AB that includes consulting or advisory. Dr. Göran Rådegran reports a personal relationship with Actelion Pharmaceuticals Sweden AB, Bayer Health Care, GlaxoSmithKline, Janssen Cilag AB and Nordic Infucare that includes consulting or advisory. Dr Rådegran is and has been primary-, or co-, investigator in; clinical PAH trials for Acceleron, Actelion Pharmaceuticals Sweden AB, Bayer, GlaxoSmithKline, Janssen-Cilag AB, MSD, Pfizer and United Therapeutics, and in clinical heart transplantation immunosuppression trials for Novartis.
